# Geriatric benign paroxysmal positional vertigo: a single-center study

**DOI:** 10.1016/j.bjorl.2023.101277

**Published:** 2023-06-03

**Authors:** Ning Song, Yuexia Wu, Xiang Li, Qianqian Wang, Xinyan Ma, Xu Yang

**Affiliations:** Peking University Aerospace School of Clinical Medicine, Aerospace Center Hospital, Department of Neurology, Peking, China

**Keywords:** Benign paroxysmal positional vertigo, Geriatric, Cupulolithiasis, Canalith repositioning procedure

## Abstract

**Objective:**

To compare the clinical features, risk factors, distribution of Benign Paroxysmal Positional Vertigo (BPPV) subtypes, and effectiveness of canalith repositioning between geriatric and non-geriatric patients with BPPV.

**Methods:**

A total of 400 patients with BPPV were enrolled. Canalith repositioning was performed according to the semicircular canals involved. Patients were divided by age into a geriatric group (≥60 years) and a non-geriatric group (20–59 years). Clinical characteristics, potential age-related risk factors, distribution of subtypes, and effectiveness of canalith repositioning were compared between the groups.

**Results:**

Female sex was significantly more common in all age groups, with a peak female-to-male ratio of 5.1:1 in the group aged 50–59 years. There was a higher proportion of men in the geriatric group. A history of disease associated with atherosclerosis was significantly more common in the geriatric group (*p* < 0.05). Migraine was significantly more common in the non-geriatric group (*p* = 0.018), as was posterior canal BPPV. The horizontal canal BPPV (especially horizontal canal BPPV-cupulolithiasis), and multicanal BPPV subtypes were more common in the geriatric group, whereas anterior canal BPPV was more common in the non-geriatric group. Two canalith repositioning sessions were effective in 58.0% of the geriatric cases and in 72.6% of the non-geriatric cases (*p* = 0.002). There was a tendency for the effectiveness of canalith repositioning to decrease with increasing age.

**Conclusion:**

BPPV was more common in women. However, the proportion of men with BPPV increased with age. Elderly patients often had a history of diseases associated with atherosclerosis (i.e., hypertension, diabetes, and hyperlipidemia). The horizontal canal BPPV (particularly horizontal canal BPPV-cupulolithiasis) and multicanal BPPV subtypes were more common and the anterior canal BPPV subtype was less common in elderly patients. The effectiveness of canalith repositioning may decrease with age. Therefore, older patients should receive more comprehensive medical treatment.

**Level of evidence:**

4.

## Highlights

• Benign Paroxysmal Positional Vertigo (BPPV) was more common in women.
• The proportion of men with BPPV increased with age.
• Geriatric BPPV patients often had risk factors associated with atherosclerosis.
• Horizontal canal-BPPV and multi-canal-BPPV were more common in geriatric patients.
• The effectiveness of canalith repositioning procedure may decrease with age.

## Introduction

Populations around the world are now aging at a rapid pace, with an increase in the numbers and proportions of individuals aged 60 years or older. Dizziness and imbalance are two of the most common complaints in the elderly. The prevalence of these symptoms ranges from approximately 20%–30% and has a tendency to increase with age.^1^, ^2^ Benign Paroxysmal Positional Vertigo (BPPV) is one of the most common causes.^3^, ^4^ An as yet unpublished study of the etiologic distribution of dizziness and vertigo in our neurology clinic showed that BPPV accounted for these symptoms in about 46.0% of elderly patients. A population-based study^5^ found that the prevalence of BPPV was 3.4% in individuals over the age of 60 years and that the 1-year prevalence was almost 7 times that in people under 40 years of age. Elderly patients with BPPV may experience more severe dizziness/vertigo and unsteadiness and have a higher risk of falls, which can limit their ability to perform everyday activities.

About 95% of cases of BPPV are idiopathic or degenerative, and aging and degenerative changes are responsible for the high incidence of BPPV in the elderly.^6^ With increasing age, there is a decrease in the amount of calcium carbonate crystals in the otoliths. This demineralization process weakens the fibrous connections between the otoliths, resulting in fissures or fragmentation, less stable otoliths, and dislodgement of otoconia in the utricles into the semicircular canals.^6^ Furthermore, elderly patients often arrive to hospital late, have a longer duration of illness, and are likely to have chronic comorbidities, such as hypertension, diabetes, and osteoporosis, which may be risk factors for BPPV and have a significant effect on their clinical presentation and prognosis. Previous studies^7^ have shown that the Canalith Repositioning Procedure (CRP) is less effective in elderly patients with BPPV than in younger patients. Moreover, the incidence of residual symptoms after a CRP, such as prolonged episodes of dizziness and unsteadiness, increases significantly with advancing age.^8^ These residual symptoms may even progress to chronic dizziness or secondary persistent postural-perceptual dizziness^9^ and cause emotional distress in older people.^10^ Therefore, BPPV may affect elderly patients in multiple dimensions, including lifestyle, social behavior, and psychosocial aspects,^11^ and is not conducive to healthy aging. Furthermore, diagnosis and treatment of BPPV may be difficult in older people. However, little attention has been paid to the elderly population with BPPV.

In this study, we compared the clinical characteristics of and risk factors for BPPV, the distribution of its subtypes, and the effectiveness of CRPs between elderly and non-elderly patients in an effort to improve the diagnosis and treatment of BPPV in the geriatric population.

## Methods

### Patients

This study had a retrospective design and included 400 patients who were diagnosed with BPPV in the Vertigo and Neurology Clinic at Aerospace Center Hospital between January 2018 and January 2021. In all cases, BPPV was diagnosed based on the Bárány Society criteria,^8^ namely, presence of nystagmus on the Dix-Hallpike, Supine-Roll, and straight head hanging tests. Nystagmus was recorded using a videonystagmography system (Interacoustics, Assens, Denmark), and therapeutic diagnosis was performed when necessary. Two CRPs were performed according to the semicircular canals involved. Reexamination by positional testing were performed after at least 1 h of rest on the day of treatment or on the following day. The treatment was considered successful if vertigo and nystagmus had disappeared at the time of the post-treatment evaluation. Magnetic resonance imaging of the brain was performed in some patients with refractory BPPV to exclude a central nervous system disorder. The subjects were divided into a geriatric group (aged ≥60 years) and a non-geriatric group (aged 20–59 years) and compared for distribution of sex and BPPV subtypes and the effectiveness of the CRPs. One hundred and ninety-seven of the 400 patients had complete clinical data available and were evaluated further for their history of dizziness/vertigo, migraine, hypertension, diabetes, hyperlipidemia, coronary heart disease, cerebrovascular disease, and motion sickness, results of auditory evaluations and caloric tests, and potential age-related risk factors and underlying mechanisms.

This study was conducted according to the guidelines of the Declaration of Helsinki and approved by the Ethics Committee of Peking University Aerospace School of Clinical Medicine (Aerospace Center Hospital) (No. 2022-033). Signed informed consent was obtained from all patients.

### Statistical analysis

Continuous variables were presented as mean ± Standard Deviation (SD). Categorical variables were expressed as percentages. The Chi-Square test was used to compare the differences between groups, with Yate’s continuity correction or Fisher's exact test applied if necessary. All tests were two-sided, and a *p*-value of <0.05 was considered statistically significant. Data analyses were performed using the SPSS software package (version 26.0, IBM Corp., Armonk, NY, USA).

## Results

### Patient demographics and clinical characteristics

**Figure 1:**
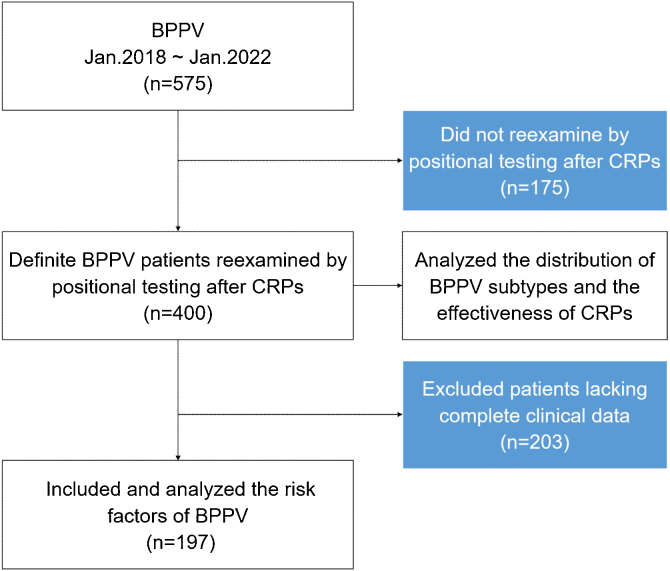
Flow chart showing the process used to select patients for inclusion in this retrospective study. BPPV, Benign Paroxysmal Positional Vertigo; CRPs, Canalith Repositioning Procedures; VNG, Videonystagmography.

**Figure 2:**
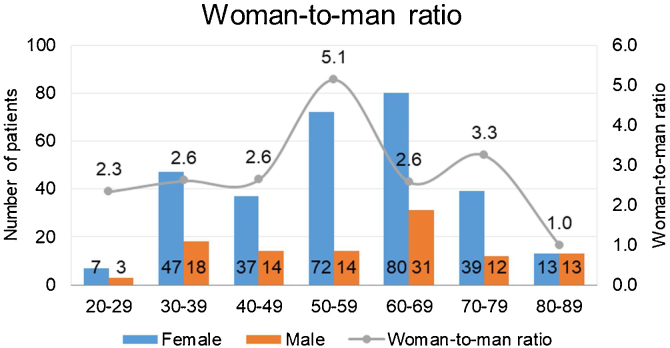
Female-to-male ratio in 400 patients with benign paroxysmal positional vertigo.

A flow chart summarizing the study enrollment process is shown in Fig. 1. Four hundred patients with a diagnosis of BPPV were retrospectively evaluated. There were 188 patients (47.0%) in the geriatric group (mean age, 69.52 ± 7.27 years) and 212 (53.0%) in the non-geriatric group (mean age, 44.67 ± 9.96 years). The female-to-male ratio was 2.4:1 (132:56) in the geriatric group and 3.3:1 (163:49) in the non-geriatric group (Table 1). The female-to-male ratio was stratified further by age, as shown in Fig. 2.

**Table 1:** Demographic characteristics of patients included in the study.

Demographic characteristics	Geriatric group	Non-geriatric group
Number	188 (47.0%)	212 (53.0%)
Age (mean, range, ± SD, years)	69.52 (60–87) ± 7.27	44.67 (20–59) ± 9.96
Sex ratio (female: male)	132 (70.2%):56 (29.8%)	163 (76.9%):49 (23.1%)

### Risk factors

Risk factors were investigated in the 197 patients for whom complete clinical data were available (geriatric group, *n* = 78; non-geriatric group, *n* = 119; Table 2). The incidence rates for history of dizziness/vertigo, hypertension, diabetes mellitus, hyperlipidemia, coronary heart disease, and cerebrovascular disease were significantly higher in the geriatric group than in the non-geriatric group (*p* < 0.05, Chi-Squared test). The incidence of migraine was significantly higher in the non-geriatric group (14.1% [11/78] vs. 28.6% [34/119], *p* = 0.018, Chi-Squared test). There was no significant difference in the incidence of motion sickness between the geriatric and non-geriatric groups (15.4% [12/78] vs. 16.0% [19/119], *p* = 0.913, Chi-Squared test). Precipitating factors before onset of BPPV were reported significantly more often in the non-geriatric group than in the geriatric group (38.7% [46/119] vs. 17.9% [14/78], *p* = 0.002, Chi-Squared test). Precipitating factors in the non-geriatric group included fatigue (16.0%, *n* = 19), poor sleep (13.4%, *n* = 16), mood swings (8.4%, *n* = 10), head trauma (3.4%, *n* = 4), and an infectious episode (2.5%, *n* = 3).

**Table 2:** Risk factors for benign paroxysmal positional vertigo in the geriatric and non-geriatric groups.

Variables	Geriatric group (*n* = 78)	Non-geriatric group (*n* = 119)	*p*-value	χ^2^ value
Previous history				
Hypertension	44 (56.4%)	14 (11.8%)	0.000*	45.209
Diabetes mellitus	21 (26.9%)	8 (6.7%)	0.000*	15.315
Hyperlipidemia	20 (25.6%)	17 (14.3%)	0.046*	3.983
Coronary heart disease	17 (21.8%)	5 (4.2%)	0.000*	14.701
Cerebrovascular disease	13 (16.7%)	3 (2.5 %)	0.000*	12.634
Migraine	11 (14.1%)	34 (28.6%)	0.018*	5.597
Motion sickness	12 (15.4%)	19 (16.0%)	0.913	0.012
Precipitating factor	14 (17.9%)	46 (38.7%)	0.002*	9.538
Fatigue	6 (7.7%)	19 (16.0%)		
Sleep disorder	5 (6.4%)	16 (13.4%)		
Mood swings	3 (3.8%)	10 (8.4%)		
Stress	0 (0)	8 (6.7%)		
Head trauma	1 (1.3%)	4 (3.4%)		
Preceding infection	1 (1.3%)	3 (2.5%)		

* p < 0.05

### Auditory and vestibular function

Symptoms such as hearing loss, ear congestion, and tinnitus were more common in the geriatric group than in the non-geriatric group (62.8% [49/78] vs. 34.5% [41/119], *p* < 0.001). When the caloric test was performed to evaluate the function of the horizontal canals, there was no significant difference in the rate of abnormal caloric tests between the geriatric and non-geriatric groups (29.5% [23/78] vs. 30.3% [36/119]; *p* > 0.05; Table 3).

**Table 3:** Results of auditory and vestibular function evaluation in the geriatric and non-geriatric groups.

Variables	Geriatric group (*n* = 78)	Non-geriatric group (*n* = 119)	*p*-value	χ^2^ value
Auditory symptom	49(62.8%)	41(34.5%)	0.000*	15.279
Hearing loss	26(33.4%)	18(15.1%)	0.003*	9.004
Ear stuffiness	12(15.4%)	19(16.0%)	0.913	0.012
Tinnitus	38(48.7%)	33 (27.7%)	0.003*	9.003
Abnormal caloric test results	23(29.5%)	36(30.3%)	0.909	0.013

* p < 0.05

### Distribution of subtypes

Posterior Canal (PC)-BPPV was the most common subtype in both groups but was significantly more common in the non-geriatric group (65.1% [137/212] vs. 51.6% [97/188], *p* = 0.008, Chi-Squared test). The proportion of patients with horizontal canal (HC)-BPPV in the geriatric group was higher than that in the non-geriatric group (30.4% [60/188] vs. 18.9% [40/212], *p* = 0.003); in particular, the proportion of patients with HC-BPPV-cupulolithiasis (HC-BPPV-cu) was higher in the geriatric group than in the non-geriatric group (16.0% [30/188] vs. 6.0% [13/212], *p* = 0.002). The proportion of patients with Multicanal (MC)-BPPV was significantly higher in the geriatric group (9.0% [17/188] vs. 1.9% [4/212], *p* = 0.001). However, the proportion of patients with Anterior Canal (AC)-BPPV was higher in the non-geriatric group (7.4% [14/188] vs. 14.6% [31/212], *p* = 0.023; Table 4).

**Table 4:** Distribution of BPPV subtypes in the geriatric and non-geriatric groups.

BPPV subtypes	Geriatric group (*n* = 188)	Non-geriatric group (*n* = 212)	*p*-value
PC-BPPV	97 (51.6%)	137 (64.6%)	0.008*
PC-BPPV-ca	69 (36.7%)	98 (46.2%)	0.054
PC-BPPV-cu	28 (14.9%)	39 (18.4%)	0.349
HC-BPPV	60 (31.9%)	40 (18.9%)	0.003*
HC-BPPV-ca	30 (16.0%)	27 (12.7%)	0.358
HC-BPPV-cu	30 (16.0%)	13 (6.0%)	0.002*
AC-BPPV	14 (7.4%)	31 (14.6%)	0.023*
MC-BPPV	17 (9.0%)	4 (1.9%)	0.001*

BPPV, Benign Paroxysmal Positional Vertigo; PC, Posterior Canal; ca, Canalolithiasis; cu, Cupulolithiasis; HC, Horizontal Canal; AC, Anterior Canal; MC, Multiple Canals.

* p < 0.05

### Effectiveness of the CRPs

**Figure 3:**
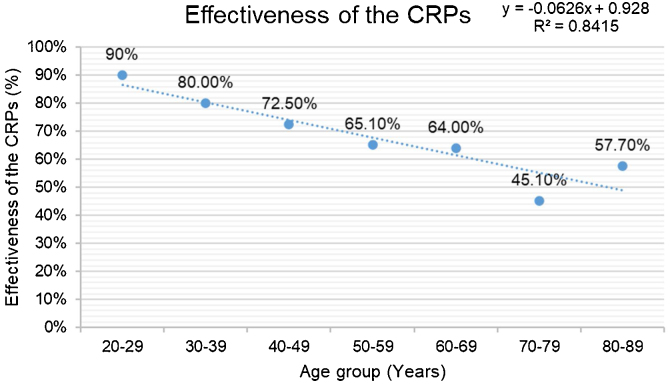
Effectiveness of the canalith repositioning procedure (*n* = 400).

Reexamination by positional testing after treatment revealed that the CRPs were significantly less likely to be effective in the geriatric group than in the non-geriatric group (58.0% [109/188] vs. 72.6% [154/212], *p* = 0.002). Furthermore, stratification of the results by age showed that the effectiveness of the CRPs tended to decrease with advancing age (Fig. 3).

## Discussion

This study had two important findings concerning the demographic features of BPPV. First, BPPV was more common in women in all age groups and particularly so in the perimenopausal period, which is in line with previous reports.^9^ The female-to-male ratio was highest (4.4:1) in patients aged 50–60 years. Considering that menopause usually occurs at the age of 45–55 years, the high incidence of BPPV in women aged 50–60 years may be related to fluctuations or deficiency of estrogen in the perimenopausal and postmenopausal periods, which could lead to worse degeneration of otoliths in elderly women. Previous studies have confirmed that estrogen replacement therapy significantly reduces the incidence of BPPV in perimenopausal women.^10^ Second, there was a high proportion of men with BPPV in the group aged ≥80 years, which again is consistent with the findings of previous research.^9^ Middle-aged and elderly men may also develop Late-Onset Hypogonadism (LOH) in response to decreasing levels of androgens such as testosterone, which is more significant after the age of 60 years. A longitudinal study in Baltimore^12^ found a significant increase in LOH in men after this age. LOH and concomitant hyperlipidemia, diabetes, hypertension, and obesity are gradually becoming recognized as having a common pathophysiological basis. In this study, hypertension, diabetes, hyperlipidemia, cardiovascular disease, and cerebrovascular disease were significantly more common in geriatric patients than in non-geriatric patients, which is in line with previous research.^13^ LOH in men can also lead to decreased bone mineral density and osteoporosis, which may contribute to the increased incidence of BPPV in elderly men. Contrasting with relevant research into women, there are still few studies on the underlying pathogenesis of BPPV in older men and the reasons for the decrease in the female-to-male ratio with advancing age, especially in the group aged 80–89 years. More studies of the pathophysiology of BPPV in older men are needed in the future.

We also investigated several putative risk factors for BPPV, including history of diseases associated with atherosclerosis, such as hypertension, diabetes, and hyperlipidemia, and found that the prevalence of these diseases was much higher in geriatric patients than in non-geriatric patients. Cardiovascular and cerebrovascular diseases are not uncommon in the geriatric age group, and their prevalence in geriatric patients with BPPV has been reported to be significantly higher than that in the general elderly population in China,^14^ which suggests that the presence of vascular disease may worsen overall health status, including the vestibular system. Vascular risk factors may affect the microcirculation in the inner ear and the metabolic balance of calcium in the otoliths in the utricle, leading to an increased risk of episodes of BPPV. The blood supply to the labyrinth is via the internal auditory artery, which usually originates from the anteroinferior cerebellar artery.^15^ The internal auditory artery is a terminal vessel and does not have collateral anastomotic connections that would allow continuation of a blood supply to the end organs if an ischemic event occurred, which would render the labyrinth especially vulnerable to ischemic phenomena.^16^ Moreover, the cochlea is adjacent to the vestibular system and supplied by the same artery; therefore, it would be easy for these structures to be damaged simultaneously. We found that 63% of patients in our geriatric group reported auditory symptoms, such as hearing loss, tinnitus, or ear congestion. This finding is consistent with the results of a previous study,^17^ indicating that the incidence of tinnitus symptoms is significantly higher in geriatric patients with BPPV than in the general elderly population.

In this study, non-geriatric patients were more likely to have precipitating factors other than arteriosclerosis. Approximately 29% of our non-geriatric patients had a history of migraine, which was significantly higher than the proportion in geriatric patients. Previous research has shown that close to half (47%) of patients in whom the onset of BPPV is before the age of 50 years have a history of migraine,^18^ and the prevalence of migraine is known to be high in women. Faralli et al.^19^ found that the mean age of onset of BPPV was 39 ± 9.2 years in patients with migraine and 53 ± 7.3 years in those without migraine, which suggests that the onset of BPPV is earlier in migraineurs. The mechanism for BPPV in younger patients may involve repeated vasospasm or a microvascular vestibulocochlear disorder, which results in damage to the vestibular epithelium and movement of dislodged otoliths from the utricle into the semicircular canal. Vasospasm in the labyrinthine system that causes vestibular damage and recurrent BPPV may be similar to the vasospasm that occurs in the ophthalmic artery, which leads to the visual disturbances associated with migraine.^18^, ^19^, ^20^ However, no direct pathophysiological link has been established between migraine and BPPV. Another finding in our study was that episodes of BPPV in the non-geriatric group were more likely to be preceded by fatigue, heavy pressure of work, poor sleep, mood swings, stress, or an infectious episode. These stressors may cause transient damage to the inner ear. A report by Monzani et al.^21^ suggested that negative life events could interact with labyrinthine function and cause abnormal activation of the hypothalamic-pituitary-adrenal axis and an increase in stress-related hormones, which may interfere with blood flow and the balance of fluid in the inner ear, affecting in particular endolymphatic calcium metabolism in the otoliths. The specific mechanism(s) of the interaction between stress hormones and development of BPPV should be investigated further.

Previous studies have shown that PC-BPPV is the most frequent subtype of BPPV in both the general population and geriatric patients.^22^, ^23^, ^24^ In our study, HC-BPPV and MC-BPPV were more common in the geriatric group than in the non-geriatric group, and especially HC-BPPV-cu, which accounted for 50% of cases of HC-BPPV. BPPV in the geriatric group often involved multiple canals, where a large number of otoliths were dislodged into more than one canal. An age-related decline in utricular function (indicated by the oVEMP amplitude) has also been reported after the age of 60–80 years.^25^ A possible explanation for this finding could be an age-related decrease in the number of calcium carbonate crystals, which leads to weakening of the fibrous connections between the otoliths. When the dislodged utricular otoconia moves into the semicircular canals and attaches to the cupula, the cupula deflects in response to the otoconial fragments. This subtype is known as cupulolithiasis and is consistent with the classical theory proposed by Schuknecht.^26^ Si et al.^27^ found that ageotropic positional nystagmus was more likely to be accompanied by atherosclerotic disease, which may also be an important cause of inner ear ischemia and deteriorating function of the utricles. Nahm et al.^28^ suggested that the higher proportion of HC-BPPV-cu in the elderly population may be explained by the pathophysiology of ageotropic positional nystagmus rather than by otoconial attachment on the cupula in the lateral semicircular canal in the elderly; for example, alteration of the biochemical composition and density of inner ear fluid resulting from a change in serum osmolality or breakdown of the blood–labyrinth barrier function as a result of inflammation in the inner ear. This is consistent with the poor effectiveness of the CRP in patients with HC-BPPV-cu when compared with the other subtypes.^22^ However, further research is needed to confirm this hypothesis.

The effectiveness of the CRP was significantly higher in our non-geriatric group but tended to decrease with age. Non-geriatric patients are more likely to have idiopathic BPPV and less severe auditory symptoms, and their inner ear damage is usually mild. Most of these patients recover rapidly after two CRPs. However, geriatric patients often have multiple comorbidities, including hypertension, diabetes, hyperlipidemia, cardiovascular disease, cerebrovascular disease, and complications that are risk factors for recurrent attacks of BPPV.^29^ In our present study, geriatric patients with BPPV often had auditory symptoms, such as hearing loss, ear congestion and tinnitus, indicating that this group may have more severe inner ear damage, which could be easily overlooked during the diagnosis and treatment process. Furthermore, a large proportion of elderly patients have the HC-BPPV-cu and MC-BPPV subtypes, which may respond less well to treatment. All of the abovementioned factors may lead to poor effectiveness of treatment, recurrent vertigo, and slow recovery in geriatric patients. Therefore, comprehensive treatment that includes therapy for atherosclerosis and other potential risk factors is needed in geriatric patients with BPPV to improve the circulation in the inner ear. At the same time, elderly patients should receive safety education to reduce their risk of falls and advice on dealing with mood swings to help improve their quality of life, and thus promote sustainable healthy aging.

### Limitations

First, this study had a retrospective design and included a relatively small sample size from one center. Therefore, the possibility of sampling bias cannot be excluded, and our findings need to be validated in a larger sample. Second, the study did not include a healthy control group. In a future study, we will include healthy controls, divide geriatric and non-geriatric patients with BPPV according to the absence or presence of risk factors, and use multiple logistic regression to explore (1) Whether the results observed are independent of age, (2) Whether there are differences in the occurrence of the different types of BPPV and the effectiveness of CRPs between geriatric and non-geriatric BPPV patients with and without risk factors, and (3) whether there is any age-related difference in occurrence of BPPV after exposure to the same risk factor for the same length of time. Furthermore, longer-term follow-up is needed to observe patterns of recurrence of BPPV and recovery according to age group.

## Conclusion

BPPV is more common in women, especially those who are perimenopausal. However, the proportion of men with BPPV increases with age. Geriatric patients with BPPV are more likely to have risk factors associated with atherosclerosis, such as hypertension, diabetes, and hyperlipidemia, which may affect the blood supply to the inner ear. The HC-BPPV (especially HC-BPPV-cu) and MC-BPPV subtypes are more common in geriatric patients. The effectiveness of CRPs may decrease with age. Therefore, more comprehensive treatment that includes management of risk factors is needed in geriatric patients with BPPV.

## Authors’ contributions

Xu Yang contributed to the conception and design of the study. Xiang Li, Xinyan Ma, Qianqian Wang collected the clinical data. Ning Song, Yuexia Wu analyzed the results and drafted and corrected the manuscript. All authors contributed to the article and approved the submitted version.

## Funding

This study was supported by Aerospace Center Hospital (Grant No. YN202106).

## Conflicts of interest

The authors declare no conflicts of interest.
